# Automatic Segmentation of Kidneys and Kidney Tumors: The KiTS19 International Challenge

**DOI:** 10.3389/fdgth.2021.797607

**Published:** 2022-01-04

**Authors:** Niranjan J. Sathianathen, Nicholas Heller, Resha Tejpaul, Bethany Stai, Arveen Kalapara, Jack Rickman, Joshua Dean, Makinna Oestreich, Paul Blake, Heather Kaluzniak, Shaneabbas Raza, Joel Rosenberg, Keenan Moore, Edward Walczak, Zachary Rengel, Zach Edgerton, Ranveer Vasdev, Matthew Peterson, Sean McSweeney, Sarah Peterson, Nikolaos Papanikolopoulos, Christopher Weight

**Affiliations:** ^1^Department of Urology, University of Minnesota, Minneapolis, MN, United States; ^2^Department of Computer Science & Engineering, University of Minnesota, Minneapolis, MN, United States; ^3^Department of Urology, University of North Dakota, Grand Forks, ND, United States; ^4^Department of Undergraduate Studies, Carleton College, Northfield, MN, United States; ^5^Department of Undergraduate Studies, Brigham Young University, Provo, UT, United States

**Keywords:** kidney tumors, semantic segmentation, medical images, renal mass, ct scans

## Abstract

**Purpose:** Clinicians rely on imaging features to calculate complexity of renal masses based on validated scoring systems. These scoring methods are labor-intensive and are subjected to interobserver variability. Artificial intelligence has been increasingly utilized by the medical community to solve such issues. However, developing reliable algorithms is usually time-consuming and costly. We created an international community-driven competition (KiTS19) to develop and identify the best system for automatic segmentation of kidneys and kidney tumors in contrast CT and report the results.

**Methods:** A training and test set of CT scans that was manually annotated by trained individuals were generated from consecutive patients undergoing renal surgery for whom demographic, clinical and outcome data were available. The KiTS19 Challenge was a machine learning competition hosted on grand-challenge.org in conjunction with an international conference. Teams were given 3 months to develop their algorithm using a full-annotated training set of images and an unannotated test set was released for 2 weeks from which average Sørensen-Dice coefficient between kidney and tumor regions were calculated across all 90 test cases.

**Results:** There were 100 valid submissions that were based on deep neural networks but there were differences in pre-processing strategies, architectural details, and training procedures. The winning team scored a 0.974 kidney Dice and a 0.851 tumor Dice resulting in 0.912 composite score. Automatic segmentation of the kidney by the participating teams performed comparably to expert manual segmentation but was less reliable when segmenting the tumor.

**Conclusion:** Rapid advancement in automated semantic segmentation of kidney lesions is possible with relatively high accuracy when the data is released publicly, and participation is incentivized. We hope that our findings will encourage further research that would enable the potential of adopting AI into the medical field.

## Introduction

Imaging technology has enhanced the diagnosis of renal masses as cross-sectional abdominal imaging has become a prominent procedure (1). These scans are mostly performed for non-urological indications and subsequently the majority of newly diagnosed renal masses are incidental. Once the abnormality is identified, specialists generally rely on imaging characteristics to assess the malignancy potential of the mass and the subsequent treatment strategy. Nephrometry scores were designed to help quantify the complexity of the mass and help guide surgical treatment, prognosis, and patient decision-making. In the current clinical practice, nephrometry scores are manually calculated using the methods developed by author Kutikov and Uzzo. These scores are calculated using data/measurements from cross-sectional imaging (2). However, the measurements require additional unreimbursed time from clinicians who already work incessantly, which subjects the scores to considerable interobserver variability. Therefore, widespread usage has been limited despite the potential clinical benefit.

Advances in artificial intelligence (AI), specifically the success of deep learning algorithms to correctly classify or “interpret” images, have given rise to nascent applications in the biomedical field (3). Renal tumors have the tendency to image well, as they are distinguishable on CT scan from the kidney parenchyma at diameters as small as 10 mms, which allows them to have the potential to become fully delineated automatically through deep learning. The AI-generated segmentation can be used with codes to translate the segmentations into fully automated nephrometry scores. Its consistency would aid clinicians to make quality decisions regarding patient care. Initial forays into this realm are also being explored in other solid tumors and anatomical regions of interest (4). There has already been initial work in this field using CT texture analysis to try to differentiate angiomyolipomas (a subtype of benign renal masses) from malignant tumors (5–7). However, texture analysis often relies on expert segmentation to extract features that are meant to be discriminative, and hence requires considerable error-prone manual effort (8). Reliable automatic segmentation of kidneys and kidney tumors would alleviate the labor-intensive process of manual segmentation which is one of the primary obstacles limiting the translation of these technologies into the clinic.

This paper describes an international community-driven competition to develop and identify the best system for automatic segmentation of kidneys and kidney tumors in contrast CT. More than 100 competing teams developed systems based on a large collection of imaging studies with high-quality manual segmentations made available to them in the spring of 2019. Teams then submitted their systems' predictions on a hold-out set of cases for which the manual segmentations were undisclosed. These predictions were centrally aggregated and scored according to a widely used agreement metric. The purpose of this paper is to characterize the performance of these automated solutions and how it relates to tumor characteristics. Our hope is that this will enable a heightened awareness of the potential weaknesses of automatic segmentation systems and help to guide their future development.

## Methods

### Dataset

**Figure 1:**
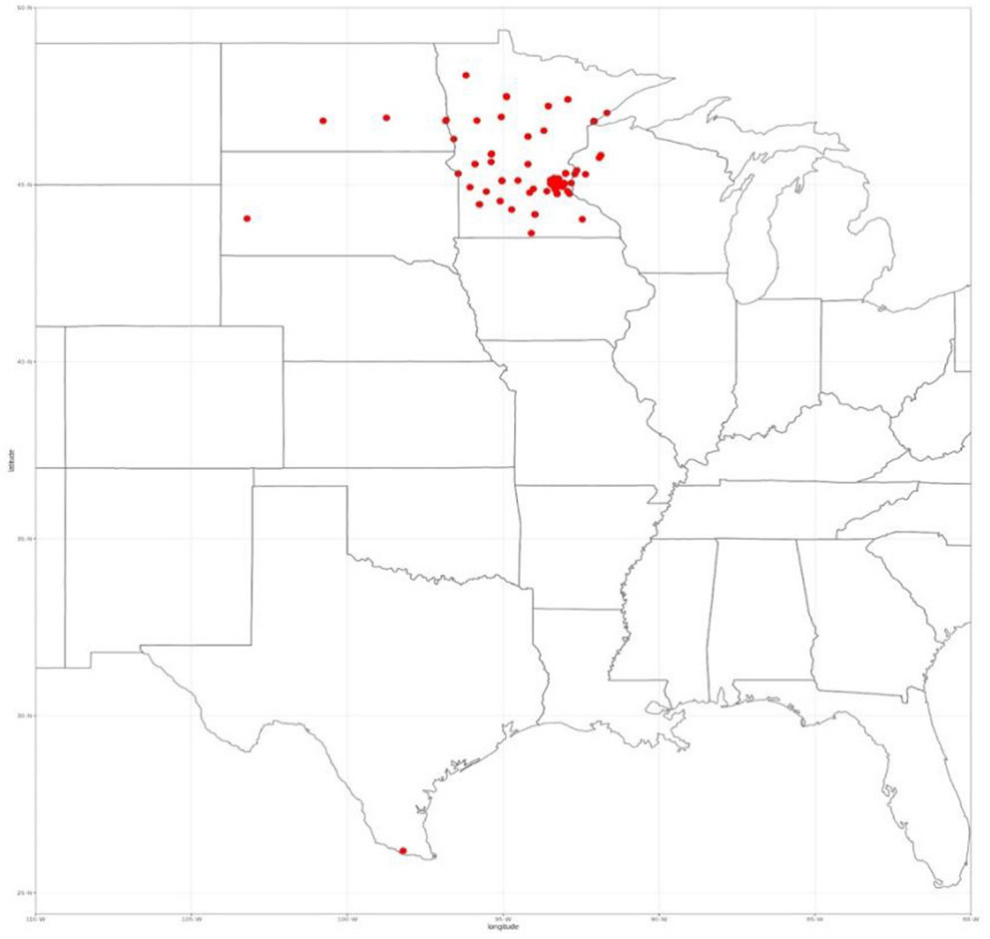
Referral sites of the different locations where the imaging was performed.

All patients who underwent surgery for a renal mass between January 2010–July 2018 were eligible for inclusion (*n* = 544) as the state of Minnesota is an opt-out state, meaning that each patient that attended our clinic is asked to sign a HIPAA form that enables them to share their data for research. They must actively opt-out if they wish their data not to be shared. Our Clinical Data Repository (CDR) office pulled the data after obtaining approval from our Institutional Review Board (IRB) for consent waivers for this retrospective study. The CDR office ensured that only patients who signed the HIPAA form consenting to have their data used in research are accessible via the encrypted and secure data shelter hosted by the University of Minnesota. The research team then assigned each scan a random case number that prevent any protected health information from being released to the public. We restricted the inclusion to only patients with available pre-operative CT abdominal/pelvic imaging in the late arterial phase (*n* = 326). The images were acquired at over 70 different clinics with scanners spanning four different manufacturers. We restricted to late-arterial phase CT images for consistency and because this was the most common contrast phase available. We also excluded patients that had a tumor thrombus to simplify an unambiguous definition of kidney tumor voxels (*n* = 26). Therefore, we included 300 patients in this study. Even though all patients underwent surgery at a single site, their images were acquired from over 70 different clinics across the country within a month before surgery, with scanners spanning four different manufactures (see Figure 1).

We reviewed the medical record of all included patients to extract pre-operative demographic and clinical data. Intra-operative data such as surgical technique, operative time, ischaemia time (in partial nephrectomy cases) and blood transfusion were also recorded. Detailed pathological data on the excised mass were also recorded including histological subtype, T stage, and ISUP grade. Finally, post-operative progress of patients was also recorded including complications, renal function, and survival.

The CT images for each of the included patients were retrieved and reviewed. Annotations were performed to delineate the kidneys and tumors in each axial view of these 300 scans. In total, more than 50,000 regions were delineated, encompassing several 100 h of effort by a group of twenty-five medical students. They had a 60 min virtual training facilitated by a Computer Science Ph.D., student (NH) who guided them through cases. For 1 week after the initial training, the students were monitored and had their performance validated against that of a staff urologic oncologist (CW). Given the different radiodensities of normal renal parenchyma, cysts, tumors, and perinephric fat, we used simple image processing techniques such as denoising and thresholding to consistently delineate the boundaries between these structures and therefore define a reliable ground truth for each case.

The interobserver agreement of the annotation process was assessed using the gold standard for calculating an average Sørenson Dice score between human annotators using the software Python. Thirty randomly selected cases were chosen and the mean Dice score for the kidney region was 0.983, while the mean Dice was 0.923 for the tumor alone. A detailed description of the validation process is reported elsewhere in Heller et al. (9).

### The 2019 Kidney and Kidney Tumor Segmentation Challenge

The KiTS19 Challenge was a machine learning competition hosted on grand-challenge.org from March 1, 2019, to October 13, 2019 and held in conjunction with the 2019 International Conference on Medical Image Computing and Computer Assisted Intervention (MICCAI) in Shenzhen, China (10). The aim of the challenge was for teams to develop a method of automatic semantic segmentation of kidneys and tumors.

A training set of 210 fully annotated cases were made publicly available 3 months prior to the testing phase, at which time a test set of 90 cases without segmentations was released, and teams were given 2 weeks to automatically segment these cases with the systems that they developed. Teams were permitted to use other publicly available data to help develop their model. They were also mandated to submit a detailed manuscript on their study methods to be eligible for the KiTS19 Challenge. Teams were allowed to make only one submission to the KiTS19 Challenge and all predictions were required to be entirely automatic with no manual intervention. The official MICCAI 2019 Leader board was released shortly after the testing phase closed. Teams were ranked based on the average Sørensen-Dice coefficient between kidney and tumor regions across all 90 test cases. A cash prize of $5000 from Intuitive Surgical was offered to the winning team to incentivize participation. The leader board has remained open following the KiTS19 Challenge and there have been 657 submissions in total at the time of writing but only the data from the official KiTS19 Challenge is included in this paper.

RENAL nephrometry score is a standardized classification system that assesses the anatomical features of a renal tumor. The components of the nephrometry score include tumor size, the proportion of the mass which is endophytic, the proximity to the collecting system, whether it is anterior or posterior and is location relative to polar lines (2). We sought to replicate the standard tumor characteristics defined by the RENAL nephrometry score through automatic segmentations and compared its accuracy to that of manual calculations. The nephrometry score was calculated by medically trained data collectors.

### Statistical Analysis

Statistical analysis was performed in R version 3.4. We used descriptive statistics to summarize data. We plotted average Sørensen-Dice coefficients for each element of the RENAL nephrometry score, including size, endophycity, nearness to collecting system, location. We used standard cut-offs for each element that was described in the original scoring system. We calculated differences using one-way analysis of variance. Statistical significance was set at *p*-value of 0.05.

## Results

### KiTS19 Challenge Participation

There were 106 unique teams from across five continents who submitted valid predictions to the challenge of which six were excluded for not meeting all submission requirements. Therefore, 100 predictions were included in the final MICCAI 2019 Leader board. The KiTS19 Challenge was recognized at MICCAI 2019 as the challenge with the greatest number of participants (11). A convenience sample of 67 teams were anonymously surveyed about their participation in this challenge. On average, teams reported spending ~170 h (SD 212 h) working on their respective models, and only 6% of teams reported working with a physician. Submissions were entirely based on deep neural networks but there were considerable differences in pre-processing strategies, architectural details, and training procedures. A complete description of the high-performing KiTS19 Challenge methodologies was reported by Heller et al. (12).

### Winning Algorithm

The top-ranking model was submitted by the German Cancer Research Center. This submission utilized three 3D U-Net architectures which is a convolutional neural network created for volumetric segmentation in biomedicine. This submission scored a 0.974 kidney Dice and a 0.851 tumor Dice resulting in 0.912 composite score. A detailed description of this algorithm and procedure of this model is outlined in Isensee and Maier-Hein (13).

### Comparison to Benchmarks

**Figure 2:**
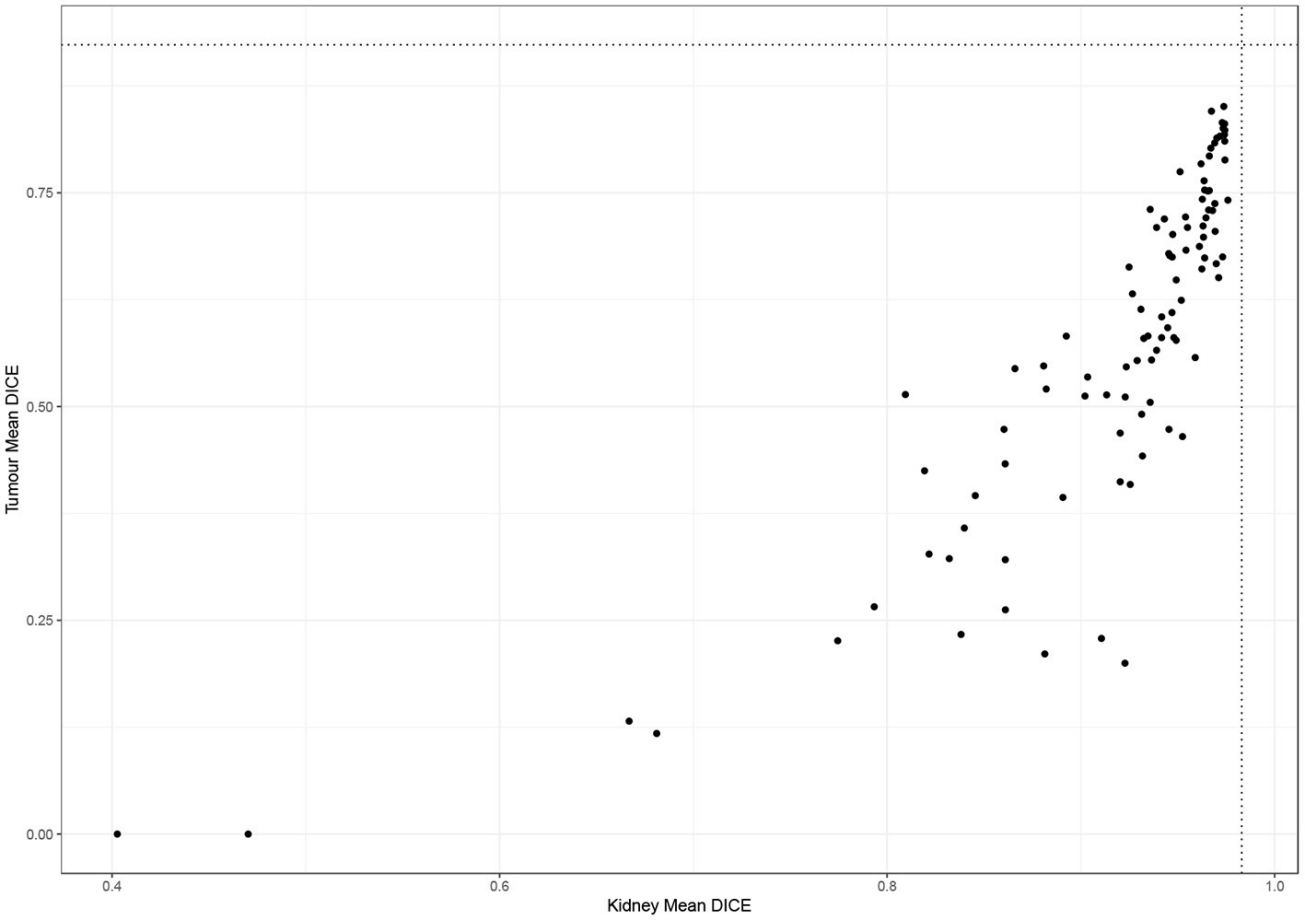
Performance of participating teams in segmenting the kidney and tumor (dotted lines represent inter-observer agreement for kidney and tumor segmentation).

Figure 2 shows the performance of all teams on the 90 test cases. Automatic segmentation of the kidney by the participating teams performed comparably to expert manual segmentation but was less reliable when segmenting the tumor. This also applied to the winning algorithm which performed well in delineating the kidney compared to the inter-observer agreement of manual segmentations (mean Dice 0.974 vs. 0.983) but was inferior to the inter-observer agreement of manual segmentations (mean Dice 0.851 vs. 0.923).

### Factors Impacting the Accuracy of Automatic Segmentation

**Figure 3:**
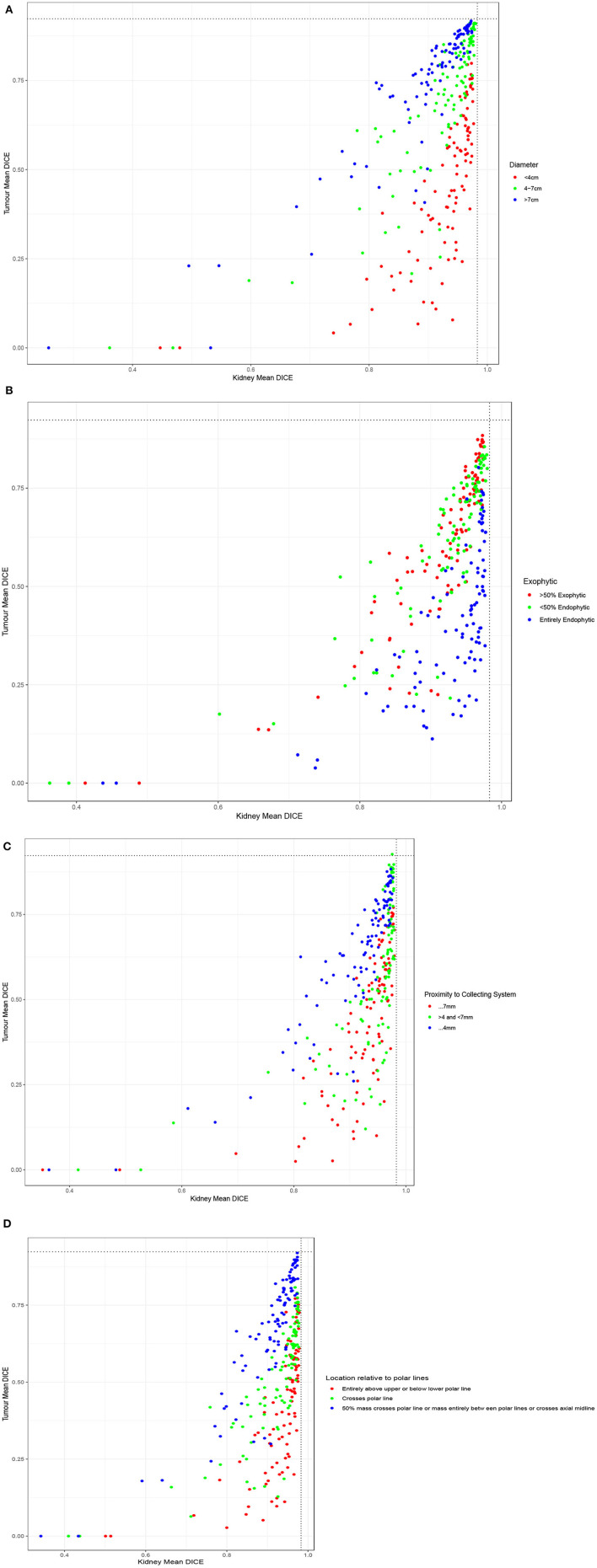
Performance of participating teams based on each component of the RENAL score: **(A)** tumor diameter, **(B)** the proportion of the mass, which is endophytic, **(C)** proximity to collecting system, and **(D)** location relative to polar lines.

When examining the components of the RENAL score, there was no significant association between kidney Dice scores and any renal components on multivariable analysis. Tumor Dice, however, significantly associated with tumor size (*p* < 0.01, Table 1A, Figure 3A), endophycity (*p* < 0.01, Table 1B, Figure 3B), collecting system involvement (*p* < 0.01, Table 1C, Figure 3C), and location relative to polar lines (*p* < 0.01, Table 1D, Figure 3D). Automatic segmentation performed worse on tumors that were smaller, more endophytic, not involved with the collecting system, and beyond the polar lines.

**Table 1:** Mean DICE for components of nephrometry score **(A)** tumor diameter, **(B)** the proportion of the mass, which is endophytic, **(C)** proximity to collecting system, and **(D)** location relative to polar lines.

**A**	**<4 cm**	**4–7 cm**	**>7 cm**	***P*-value**
Mean DICE for segmenting kidney for all teams (SD)	0.92 (0.08)	0.92 (0.10)	0.89 (0.11)	0.11
Mean DICE for segmenting tumor for all teams (SD)	0.45 (0.21)	0.70 (0.21)	0.75 (0.19)	<0.01
Winning algorithm DICE for kidney	0.97	0.97	0.97	
Winning algorithm DICE for tumor	0.80	0.91	0.89	
**B**	**>50% exophytic**	**<50% endophytic**	**Entirely endophytic**	***P*** **-value**
Mean DICE for segmenting kidney for all teams (SD)	0.91 (0.09)	0.91 (0.10)	0.92 (0.09)	0.56
Mean DICE for segmenting tumor for all teams (SD)	0.61 (0.21)	0.51 (0.20)	0.41 (0.19)	<0.01
Winning algorithm DICE for kidney	0.97	0.98	0.97	
Winning algorithm DICE for tumor	0.88	0.86	0.74	
**C**	**≥7 mm**	**>4 and** ** <7 mm**	**≤4 mm**	***P*** **-value**
Mean DICE for segmenting kidney for all teams (SD)	0.92 (0.09)	0.94 (0.09)	0.91 (0.10)	0.11
Mean DICE for segmenting tumor for all teams (SD)	0.44 (0.21)	0.56 (0.23)	0.64 (0.19)	<0.01
Winning algorithm DICE for kidney	0.97	0.98	0.97	
Winning algorithm DICE for tumor	0.75	0.93	0.88	
**D**	**Entirely above upper or below lower polar line**	**Crosses polar line**	**50% mass crosses polar line or mass entirely between polar lines or crosses axial midline**	***P*** **-value**
Mean DICE for segmenting kidney for all teams (SD)	0.93 (0.08)	0.92 (0.09)	0.90 (0.10)	0.09
Mean DICE for segmenting tumor for all teams (SD)	0.43 (0.21)	0.52 (0.19)	068 (0.20)	<0.01
Winning algorithm DICE for kidney	0.97	0.97	0.97	
Winning algorithm DICE for tumor	0.77	0.79	0.92	

## Discussion

We found that by making high-quality, segmented data publicly available and creating an incentivized challenge, we were able to coordinate ~20,000 h of effort globally on automatic segmentations of kidney and kidney tumor. This challenge demonstrated that artificial intelligence could automate the digital quantification of kidney masses with only slightly worse performance than humans, while automatic kidney segmentation was found to exhibit virtually identical performance to humans. Automated segmentation shows tremendous promise for the automation of this and related tasks. It seems certain that with additional training scans from a wider range of centers and contrast phases, the algorithms will approach and potentially surpass human level performance and make for an extremely useful tool for characterizing renal masses at point of care.

We foresee that automated segmentation will serve as a basis for many advances in prognosis, diagnosis, and the treatment of kidney tumors. Automatic segmentation could aid radiologists in flagging/identifying concerning lesions on CT scans performed for other indications and thereby reducing the risk of a missed diagnosis. Automated segmentation will also permit more widespread and unambiguous calculation of nephrometry scores such as the RENAL score (2) while incorporated components such as tumors centrality calculated by C-index (14), and the risk of surgical and medical perioperative complications calculated by PADUA scores (15) as well. While these scores have shown to be associated with a range of clinical outcomes, they have been limited by marked inter-observer variation and the human capital required to generate the scores. The inter-class correlation between radiology fellows, urology fellows, a radiology resident, and medical school students for C-index, PADUA and RENAL scores have been reported to be 0.77, 0.68, and 0.66, respectively (16). Therefore, automation of such calculations would enable the consistency of predictions that could be easily exportable even to resource-poor and medically underserved areas. Furthermore, we envision that automated segmentation of kidney tumors would open the door to sophisticated tumor and kidney analytics such as radiomics/texture and be poised to discover new imaging biomarkers associated with patient relevant outcomes. Based on imaging characteristics we may be able to predict the probability of the incidentally detected mass to be malignant or aggressive in nature. Therefore, we can use imaging characteristics to stratify risk and help guide treatment decisions.

The KiTS19 Challenge demonstrates the vast potential of community-driven efforts for developing AI applications in the medical field. Such open challenges facilitate the pooling of knowledge and efforts to identify the high-performing solutions to problems in a timely manner compared to isolated individual efforts which can take significant time and effort to replicate and benchmark against. Mak et al. conducted a financially incentivised online challenge to develop an AI solution to segment lung tumors for radiation therapy targeting (17). This challenge had 34 submitted algorithms and following multiple phases created a model with a DICE score of 0.68. This model outperformed other commercially available software and was comparable to interobserver variation between five radiation oncologists. This data suggests that additional collaborative work on the top algorithms from the KiTS19 Challenge would improve on current performance and may already be performing at a clinically acceptable level. There have also been similar competitions in other organs such as breast cancer in which the Digital Mammography DREAM Challenge attempted to segment tumors from mammograms with good success (18). The success of these events suggests that competitions in oncology can hasten the development of high-quality tools which ultimately improve outcomes for cancer patients by developing innovative methods in an open, low-cost, and swift manner.

The findings of this study should be interpreted within the context of its limitations. Firstly, the algorithms from this challenge may not function as well in a different patient population or in different ethnicities/nationalities. Despite the relatively wide range of scanners and radiology services represented in our data, they are all limited to a small geographic region. In addition, the impact of not working with a clinical expert could cause tumors to go unnoticed by the algorithms which would lead to less aggressive treatment plans such as surveillance. Furthermore, the dataset used is relatively small compared to non-segmentation or non-medical imaging AI challenges and our performance estimates are therefore less precise than we could make with a larger, more diverse dataset. Nonetheless, our dataset and the results of our challenge represent a significant advancement in kidney and kidney tumor segmentation and provide a solid platform for further improvement.

## Conclusion

Rapid advancement in automated semantic segmentation of kidney lesions is possible with relatively high accuracy when the data is released publicly, and participation is incentivized. This allows for the development of a range of clinical applications which include automated diagnosis and patient specific prediction models. These can aid in decision-making to choose the ideal treatment for cancer patients and, at the same time, facilitate better anatomy specific surgical training or planning models. The use of competitions to develop AI solutions in medicine is feasible, time-efficient, and cost-effective.

It is envisioned that reliable automatic segmentation would form the basis to quantitatively study kidney tumor morphology/texture and permit the automation of nephrometry scores and other predictors for a range of clinical outcomes (19). Therefore, segmentation is a necessary step toward creating high fidelity surgical training models such as 3D printed kidneys (20) or augmented or virtual reality.

## Data Availability Statement

The original contributions presented in the study are included in the article/supplementary materials, further inquiries can be directed to the corresponding author/s.

## Ethics Statement

The studies involving human participants were reviewed and approved by University of Minnesota IRB. The patients/participants provided their written informed consent to participate in this study.

## Author Contributions

All authors listed have made a substantial, direct, and intellectual contribution to the work and approved it for publication.

## Funding

Research reported in this publication was supported by the National Cancer Institute of the National Institutes of Health under Award Number R01CA225435.

## Author Disclaimer

The content is solely the responsibility of the authors and does not necessarily represent the official views of the National Institutes of Health.

## Conflict of Interest

The authors declare that the research was conducted in the absence of any commercial or financial relationships that could be construed as a potential conflict of interest.

## Publisher's Note

All claims expressed in this article are solely those of the authors and do not necessarily represent those of their affiliated organizations, or those of the publisher, the editors and the reviewers. Any product that may be evaluated in this article, or claim that may be made by its manufacturer, is not guaranteed or endorsed by the publisher.
